# Updated list of the mosquitoes of Colombia (Diptera: Culicidae)

**DOI:** 10.3897/BDJ.3.e4567

**Published:** 2015-03-09

**Authors:** Paula Rozo-Lopez, Ximo Mengual

**Affiliations:** ‡ Bonn University, Bonn, Germany; § Zoologisches Forschungsmuseum Alexander Koenig, Bonn, Germany

**Keywords:** Nematocera, Neotropical Region, species account, new records

## Abstract

**Background:**

A revised list of the mosquitoes (Diptera: Culicidae) known to occur in Colombia is presented. A total of 324 species from 28 genera of Culicidae are included. The species names are organized in alphabetical order according to the current generic and subgeneric classification, along with their authorship. The list is compiled in order to support mosquito research in Colombia.

**New information:**

Our systematic review and literature survey found, by 16 February 2015, 13 records of culicid species previously overlooked by mosquito catalogs for Colombia: *Anopheles costai* da Fonseca & da Silva Ramos, 1939, *An. fluminensis* Root, 1927, *An. malefactor* Dyar & Knab, 1907, *An. shannoni* Davis, 1931, *An. vargasi* Galbadón, Cova García & Lopez, 1941, *Culex mesodenticulatus* Galindo & Mendez, 1961, *Haemagogus capricornii* Lutz, 1904, *Isostomyia espini* (Martini, 1914), *Johnbelkinia leucopus* (Dyar & Knab, 1906), *Mansonia indubitans* Dyar & Shannon, 1925, *Psorophora saeva* Dyar & Knab, 1906, *Sabethes glaucodaemon* (Dyar & Shannon, 1925), and *Wyeomyia intonca* Dyar & Knab, 1909. Moreover, Wyeomyia (Dendromyia) luteoventralis Theobald, 1901 is recorded for Colombia for the first time. This work provides important insights into mosquito diversity in Colombia, using the current nomenclature and phylogenetic rankings.

## Introduction

Currently, 3,543 species of Culicidae are formally recognized and distributed over a diverse range of habitats around the world (Harbach 2015). Colombia, one of the world's megadiverse countries, has a highly diverse mosquito fauna and a high prevalence of mosquito-borne diseases (Morrone 2006, Foley et al. 2008, Hudson 2010, Montoya-Lerma et al. 2011). The tremendous variety of ecosystems in Colombia provides environmental conditions that favor the immigration, adaptation, development and persistence of a great diversity of mosquito species (Molina et al. 2000, Olano et al. 2001, Foley et al. 2007, Barreto et al. 2008, Brochero and Quiñones 2008).

Six mosquito catalogs list species that occur in Colombia: Barreto-Reyes (1955) reported 164 species; Knight and Stone (1977) reported 250 species; Carrejo and González (1992) listed 260 recognized species; Walter Reed Medical Army (1998) reported also 260 species; and lastly, 289 species were reported in the Walter ​Reed Biosystematics Unit mosquito catalog (Gaffigan et al. 2014). Recent changes in mosquito taxonomy, ranking of groups, and species names (Gaffigan et al. 2013, Harbach 2015) made it crucial to compile an updated list that follows the currently accepted classification, as well as includes new and overlooked species records for the country.

## Materials and methods

The species list was compiled from information available in previously published mosquito catalogs for Colombia (Barreto-Reyes 1955, Knight and Stone 1977, Carrejo and González 1992, Walter Reed Medical Army 1998, Gaffigan et al. 2014) and other publications containing species reports not included in these catalogs (Kumm et al. 1946, Osorno-Mesa 1947, Lane 1953, Arnell 1976, Wilkerson 1990, Pecor et al. 1992, Harbach 1995, Barreto et al. 1996, Lounibos et al. 1998, Vélez et al. 1998, Molina et al. 2000, Ferro et al. 2003, Liria and Navarro 2003, Porter and Wolff 2004, González and Carrejo 2007, Ferro et al. 2008, Harbach and Howard 2009, Mendenhall et al. 2011, Barajas et al. 2013). The first record for Colombia of Wyeomyia (Dendromyia) luteoventralis Theobald, 1901 is based on a female collected with CDC traps in September 2013 in a tropical dry forest in La Pintada (Antioquia) [N 05.63646, W 075.59002, 710m above sea level]. The specimen was identified using Lane (1953), Motta and Lourenço-de-Oliveira (1995), Judd (1998), Motta and Lourenço-de-Oliveira (2000) as references.

The species presented in this list are arranged alphabetically by genus and subgenus. Species names are followed by their authorship and year of description, and the catalog or other source that first reported the species in Colombia. Our list follows the most recent mosquito classification compiled by Gaffigan et al. (2013) and Harbach (2015). Also, we follow the provisional '*sensu auctorum*' placement of 'Ochlerotatus (Protomacleaya)', product of the polyphyletic assemblage of species reflected in the Aedini phylogeny reconstructed by Reinert et al. (2009). Junior synonyms, subspecies, biologically separate sibling species that lack formal names, and nomina dubia reported by Barreto-Reyes (1955) and Knight and Stone 1977 (*Anopheles allopha* Peryassú, 1921, *Culex aikenii* Aiken & Rowland, 1906, *Culex virgultus* Theobald, 1901, *Trichoprosopon hyperleucum* (Martini, 1931)) are not included.

## Checklists

### List of mosquito species known to occur in Colombia

#### Aedeomyia (Aedeomyia) squamipennis

(Lynch Arribálzaga, 1878)

##### Notes

Barreto-Reyes 1955

#### Anopheles (Anopheles) apicimacula

Dyar & Knab, 1906

##### Notes

Barreto-Reyes 1955

#### Anopheles (Anopheles) calderoni

Wilkerson, 1991

##### Notes

Carrejo and González 1992

#### Anopheles (Anopheles) costai

da Fonseca & da Silva Ramos, 1939

##### Notes

González and Carrejo 2007. Species previously overlooked by other mosquito catalogs for Colombia.

#### Anopheles (Anopheles) eiseni

Coquillett, 1902

##### Notes

Barreto-Reyes 1955

#### Anopheles (Anopheles) fluminensis

Root, 1927

##### Notes

González and Carrejo 2007. Species previously overlooked by other mosquito catalogs for Colombia.

#### Anopheles (Anopheles) forattinii

Wilkerson & Sallum, 1999

##### Notes

González and Carrejo 2007

#### Anopheles (Anopheles) malefactor

Dyar & Knab, 1907

##### Notes

Wilkerson 1990. Species previously overlooked by other mosquito catalogs for Colombia.

#### Anopheles (Anopheles) mattogrossensis

Lutz & Neiva, 1911

##### Notes

Barreto-Reyes 1955

#### Anopheles (Anopheles) mediopunctatus

(Lutz, 1903)

##### Notes

Barreto-Reyes 1955

#### Anopheles (Anopheles) neomaculipalpus

Curry, 1931

##### Notes

Barreto-Reyes 1955

#### Anopheles (Anopheles) peryassui

Dyar & Knab, 1908

##### Notes

Barreto-Reyes 1955

#### Anopheles (Anopheles) pseudomaculipes

(Peryassú, 1908)

##### Notes

Knight and Stone 1977

#### Anopheles (Anopheles) pseudopunctipennis

Theobald, 1901

##### Notes

Barreto-Reyes 1955

#### Anopheles (Anopheles) punctimacula

Dyar & Knab, 1906

##### Notes

Barreto-Reyes 1955

#### Anopheles (Anopheles) shannoni

Davis, 1931

##### Notes

González and Carrejo 2007. Species previously overlooked by other mosquito catalogs for Colombia.

#### Anopheles (Anopheles) vestitipennis

Dyar & Knab, 1906

##### Notes

Barreto-Reyes 1955

#### Anopheles (Kerteszia) bambusicolus

Komp, 1937

##### Notes

Barreto-Reyes 1955

#### Anopheles (Kerteszia) bellator

Dyar & Knab, 1906

##### Notes

Knight and Stone 1977

#### Anopheles (Kerteszia) boliviensis

(Theobald, 1905)

##### Notes

Barreto-Reyes 1955

#### Anopheles (Kerteszia) cruzii

Dyar & Knab, 1908

##### Notes

Knight and Stone 1977

#### Anopheles (Kerteszia) homunculus

Komp, 1937

##### Notes

Barreto-Reyes 1955

#### Anopheles (Kerteszia) lepidotus

Zavortink, 1973

##### Notes

Knight and Stone 1977

#### Anopheles (Kerteszia) neivai

Howard, Dyar & Knab, 1913

##### Notes

Barreto-Reyes 1955

#### Anopheles (Kerteszia) pholidotus

Zavortink, 1973

##### Notes

González and Carrejo 2007

#### Anopheles (Lophopodomyia) gilesi

(Peryassú, 1908)

##### Notes

Barreto-Reyes 1955

#### Anopheles (Lophopodomyia) oiketorakras

Osorno-Mesa, 1947

##### Notes

Osorno-Mesa 1947, Barreto-Reyes 1955

#### Anopheles (Lophopodomyia) squamifemur

Antunes, 1937

##### Notes

Barreto-Reyes 1955

#### Anopheles (Lophopodomyia) vargasi

Gabaldón, Cova García & Lopez, 1941

##### Notes

Osorno-Mesa 1947. Species previously overlooked by other mosquito catalogs for Colombia.

#### Anopheles (Nyssorhynchus) albimanus

Wiedemann, 1820

##### Notes

Barreto-Reyes 1955

#### Anopheles (Nyssorhynchus) albitarsis

Lynch Arribálzaga, 1878

##### Notes

Barreto-Reyes 1955

#### Anopheles (Nyssorhynchus) aquasalis

Curry, 1932

##### Notes

Barreto-Reyes 1955

#### Anopheles (Nyssorhynchus) argyritarsis

Robineau-Desvoidy, 1827

##### Notes

Barreto-Reyes 1955

#### Anopheles (Nyssorhynchus) benarrochi

Gabaldón, Cova García & Lopez, 1941

##### Notes

Barreto-Reyes 1955

#### Anopheles (Nyssorhynchus) braziliensis

Chagas, 1907

##### Notes

Barreto-Reyes 1955

#### Anopheles (Nyssorhynchus) darlingi

Root, 1926

##### Notes

Barreto-Reyes 1955

#### Anopheles (Nyssorhynchus) dunhami

Causey, 1945

##### Notes

Lounibos et al. 1998

#### Anopheles (Nyssorhynchus) evansae

Brèthes, 1926

##### Notes

Knight and Stone 1977

#### Anopheles (Nyssorhynchus) marajoara

Galvão & Damasceno, 1942

##### Notes

Knight and Stone 1977

#### Anopheles (Nyssorhynchus) nuneztovari

Gabaldón, 1940

##### Notes

Barreto-Reyes 1955

#### Anopheles (Nyssorhynchus) oswaldoi

Peryassú, 1922

##### Notes

Barreto-Reyes 1955

#### Anopheles (Nyssorhynchus) parvus

Chagas, 1907

##### Notes

Barreto-Reyes 1955

#### Anopheles (Nyssorhynchus) rangeli

Gabaldón, Cova García & Lopez, 1940

##### Notes

Barreto-Reyes 1955

#### Anopheles (Nyssorhynchus) strodei

Root, 1926

##### Notes

Barreto-Reyes 1955

#### Anopheles (Nyssorhynchus) triannulatus

Neiva & Pinto, 1922

##### Notes

Barreto-Reyes 1955

#### Anopheles (Nyssorhynchus) trinkae

Faran, 1979

##### Notes

Carrejo and González 1992

#### Anopheles (Stethomyia) kompi

Edwards, 1930

##### Notes

Barreto-Reyes 1955

#### Anopheles (Stethomyia) nimbus

(Theobald, 1902)

##### Notes

Barreto-Reyes 1955

#### Anopheles (Stethomyia) thomasi

Shannon, 1933

##### Notes

Knight and Stone 1977

#### Chagasia ablusa

Harbach, 2009

##### Notes

Harbach and Howard 2009

#### Chagasia bathana

Dyar, 1928

##### Notes

Knight and Stone 1977

#### Chagasia bonneae

Root, 1927

##### Notes

Barreto-Reyes 1955

#### Chagasia fajardi

(Lutz, 1904)

##### Notes

Barreto-Reyes 1955

#### Coquillettidia (Rhynchotaenia) albicosta

(Peryassú, 1908)

##### Notes

Barreto-Reyes 1955

#### Coquillettidia (Rhynchotaenia) arribalzagae

(Theobald, 1903)

##### Notes

Barreto-Reyes 1955

#### Coquillettidia (Rhynchotaenia) fasciolata

(Lynch Arribálzaga, 1891)

##### Notes

Carrejo and González 1992

#### Coquillettidia (Rhynchotaenia) hermanoi

(Lane & Coutinho, 1940)

##### Notes

Knight and Stone 1977

#### Coquillettidia (Rhynchotaenia) juxtamansonia

(Chagas, 1907)

##### Notes

Barreto-Reyes 1955

#### Coquillettidia (Rhynchotaenia) lynchi

(Shannon, 1931)

##### Notes

Barreto-Reyes 1955

#### Coquillettidia (Rhynchotaenia) nigricans

(Coquillett, 1904)

##### Notes

Barreto-Reyes 1955

#### Coquillettidia (Rhynchotaenia) venezuelensis

(Theobald, 1912)

##### Notes

Barreto-Reyes 1955

#### Culex flochi

Duret, 1969

##### Notes

Knight and Stone 1977.The subgeneric affiliation of this species is uncertain.

#### Culex ocellatus

Theobald, 1903

##### Notes

Knight and Stone 1977. The subgeneric affiliation of this species is uncertain.

#### Culex (Aedinus) accelerans

Root, 1927

##### Notes

Ferro et al. 2003

#### Culex (Aedinus) amazonensis

(Lutz, 1905)

##### Notes

Barreto-Reyes 1955

#### Culex (Anoedioporpa) bamborum

Rozeboom & Komp, 1948

##### Notes

Barreto-Reyes 1955

#### Culex (Anoedioporpa) browni

Komp, 1936

##### Notes

Knight and Stone 1977

#### Culex (Anoedioporpa) conservator

Dyar & Knab, 1906

##### Notes

Knight and Stone 1977

#### Culex (Anoedioporpa) corrigani

Dyar & Knab, 1907

##### Notes

Knight and Stone 1977

#### Culex (Anoedioporpa) restrictor

Dyar & Knab, 1906

##### Notes

Mendenhall et al. 2011

#### Culex (Belkinomyia) eldridgei

Adames & Galindo, 1973

##### Notes

Knight and Stone 1977

#### Culex (Carrollia) antunesi

Lane & Whitman, 1943

##### Notes

Knight and Stone 1977

#### Culex (Carrollia) bihaicola

Dyar & Núñez Tovar, 1928

##### Notes

Barreto-Reyes 1955

#### Culex (Carrollia) bonnei

Dyar, 1921

##### Notes

Barreto-Reyes 1955

#### Culex (Carrollia) infoliatus

Bonne-Wepster & Bonne, 1920

##### Notes

Barreto-Reyes 1955

#### Culex (Carrollia) kompi

Valencia, 1973

##### Notes

Knight and Stone 1977

#### Culex (Carrollia) metempsytus

Dyar, 1921

##### Notes

Barreto-Reyes 1955

#### Culex (Carrollia) secundus

Bonne-Wepster & Bonne, 1920

##### Notes

Barreto-Reyes 1955

#### Culex (Carrollia) urichi

(Coquillett, 1906)

##### Notes

Barreto-Reyes 1955

#### Culex (Carrollia) wilsoni

Lane & Whitman, 1943

##### Notes

Knight and Stone 1977

#### Culex (Culex) abnormalis

Lane, 1936

##### Notes

Knight and Stone 1977

#### Culex (Culex) acharistus

Root, 1927

##### Notes

Carrejo and González 1992

#### Culex (Culex) alani

Forattini, 1965

##### Notes

Knight and Stone 1977

#### Culex (Culex) archegus

Dyar, 1929

##### Notes

Knight and Stone 1977

#### Culex (Culex) bickleyi

Forattini, 1965

##### Notes

Knight and Stone 1977

#### Culex (Culex) bonneae

Dyar & Knab, 1906

##### Notes

Knight and Stone 1977

#### Culex (Culex) brevispinosus

Bonne-Wepster & Bonne, 1920

##### Notes

Barreto-Reyes 1955

#### Culex (Culex) camposi

Dyar, 1925

##### Notes

Knight and Stone 1977

#### Culex (Culex) chidesteri

Dyar, 1921

##### Notes

Barreto-Reyes 1955

#### Culex (Culex) chitae

Duret, 1967

##### Notes

Knight and Stone 1977

#### Culex (Culex) coronator

Dyar & Knab, 1906

##### Notes

Barreto-Reyes 1955

#### Culex (Culex) declarator

Dyar & Knab, 1906

##### Notes

Carrejo and González 1992

#### Culex (Culex) erythrothorax

Dyar, 1907

##### Notes

Gaffigan et al. 2014

#### Culex (Culex) inflictus

Theobald, 1901

##### Notes

Barreto-Reyes 1955

#### Culex (Culex) levicastilloi

Lane, 1945

##### Notes

Gaffigan et al. 2014

#### Culex (Culex) maracayensis

Evans, 1923

##### Notes

Barreto-Reyes 1955

#### Culex (Culex) mollis

Dyar & Knab, 1906

##### Notes

Barreto-Reyes 1955

#### Culex (Culex) nigripalpus

Theobald, 1901

##### Notes

Barreto-Reyes 1955

#### Culex (Culex) ousqua

Dyar, 1918

##### Notes

Knight and Stone 1977

#### Culex (Culex) quinquefasciatus

Say, 1823

##### Notes

Barreto-Reyes 1955

#### Culex (Culex) saltanensis

Dyar, 1928

##### Notes

Gaffigan et al. 2014

#### Culex (Culex) spinosus

Lutz, 1905

##### Notes

Knight and Stone 1977

#### Culex (Culex) stigmatosoma

Dyar, 1907

##### Notes

Barreto-Reyes 1955

#### Culex (Culex) thriambus

Dyar, 1921

##### Notes

Knight and Stone 1977

#### Culex (Culex) usquatissimus

Dyar, 1922

##### Notes

Knight and Stone 1977

#### Culex (Culex) usquatus

Dyar, 1918

##### Notes

Knight and Stone 1977

#### Culex (Melanoconion) adamesi

Sirivanakarn & Galindo, 1980

##### Notes

Knight and Stone 1977

#### Culex (Melanoconion) albinensis

Bonne-Wepster & Bonne, 1920

##### Notes

Knight and Stone 1977

#### Culex (Melanoconion) alogistus

Dyar, 1918

##### Notes

Knight and Stone 1977

#### Culex (Melanoconion) bastagarius

Dyar & Knab, 1906

##### Notes

Barreto-Reyes 1955

#### Culex (Melanoconion) batesi

Rozeboom & Komp, 1948

##### Notes

Barreto-Reyes 1955

#### Culex (Melanoconion) caudelli

(Dyar & Knab, 1906)

##### Notes

Knight and Stone 1977

#### Culex (Melanoconion) comatus

Senevet & Abonnenc, 1939

##### Notes

Barreto-Reyes 1955

#### Culex (Melanoconion) commevynensis

Bonne-Wepster & Bonne, 1920

##### Notes

Barreto-Reyes 1955

#### Culex (Melanoconion) comminutor

Dyar, 1920

##### Notes

Barreto-Reyes 1955

#### Culex (Melanoconion) conspirator

Dyar & Knab, 1906

##### Notes

Barreto-Reyes 1955

#### Culex (Melanoconion) coppenamensis

Bonne-Wepster & Bonne, 1920

##### Notes

Carrejo and González 1992

#### Culex (Melanoconion) creole

Anduze, 1949

##### Notes

Carrejo and González 1992

#### Culex (Melanoconion) crybda

Dyar, 1924

##### Notes

Barreto-Reyes 1955

#### Culex (Melanoconion) distinguendus

Dyar, 1928

##### Notes

Knight and Stone 1977

#### Culex (Melanoconion) dunni

Dyar, 1918

##### Notes

Barreto-Reyes 1955

#### Culex (Melanoconion) eastor

Dyar, 1920

##### Notes

Barreto-Reyes 1955

#### Culex (Melanoconion) educator

Dyar & Knab, 1906

##### Notes

Barreto-Reyes 1955

#### Culex (Melanoconion) egcymon

Dyar, 1923

##### Notes

Pecor et al. 1992

#### Culex (Melanoconion) elevator

Dyar & Knab, 1906

##### Notes

Barreto-Reyes 1955

#### Culex (Melanoconion) epanastasis

Dyar, 1922

##### Notes

Knight and Stone 1977

#### Culex (Melanoconion) erraticus

(Dyar & Knab, 1906)

##### Notes

Barreto-Reyes 1955

#### Culex (Melanoconion) ferreri

Duret, 1968

##### Notes

Knight and Stone 1977

#### Culex (Melanoconion) garcesi

Duret, 1968

##### Notes

Knight and Stone 1977

#### Culex (Melanoconion) inadmirabilis

Dyar, 1928

##### Notes

Barreto-Reyes 1955

#### Culex (Melanoconion) inhibitator

Dyar & Knab, 1906

##### Notes

Barreto-Reyes 1955

#### Culex (Melanoconion) iolambdis

Dyar, 1918

##### Notes

Lane 1953

#### Culex (Melanoconion) johnsoni

Galindo & Mendez, 1961

##### Notes

Pecor et al. 1992

#### Culex (Melanoconion) kummi

Komp & Rozeboom, 1951

##### Notes

Knight and Stone 1977

#### Culex (Melanoconion) lucifugus

Komp, 1936

##### Notes

Knight and Stone 1977

#### Culex (Melanoconion) mesodenticulatus

Galindo & Mendez, 1961

##### Notes

Barreto et al. 1996. Species previously overlooked by other mosquito catalogs for Colombia.

#### Culex (Melanoconion) ocossa

Dyar & Knab, 1919

##### Notes

Knight and Stone 1977

#### Culex (Melanoconion) panocossa

Dyar, 1923

##### Notes

Gaffigan et al. 2014

#### Culex (Melanoconion) pedroi

Sirivanakarn & Belkin, 1980

##### Notes

Ferro et al. 2003

#### Culex (Melanoconion) phlogistus

Dyar, 1920

##### Notes

Knight and Stone 1977

#### Culex (Melanoconion) pilosus

(Dyar & Knab, 1906)

##### Notes

Barreto-Reyes 1955

#### Culex (Melanoconion) quasihibridus

Galindo & Blanton, 1954

##### Notes

Knight and Stone 1977

#### Culex (Melanoconion) rooti

Rozeboom, 1935

##### Notes

Knight and Stone 1977

#### Culex (Melanoconion) serratimarge

Root, 1927

##### Notes

Knight and Stone 1977

#### Culex (Melanoconion) spissipes

(Theobald, 1903)

##### Notes

Barreto-Reyes 1955

#### Culex (Melanoconion) sursumptor

Dyar, 1924

##### Notes

Barreto-Reyes 1955

#### Culex (Melanoconion) taeniopus

Dyar & Knab, 1907

##### Notes

Barreto-Reyes 1955

#### Culex (Melanoconion) tecmarsis

Dyar, 1918

##### Notes

Pecor et al. 1992

#### Culex (Melanoconion) theobaldi

(Lutz, 1904)

##### Notes

Knight and Stone 1977

#### Culex (Melanoconion) vomerifer

Komp, 1932

##### Notes

Knight and Stone 1977

#### Culex (Melanoconion) zeteki

Dyar, 1918

##### Notes

Knight and Stone 1977

#### Culex (Microculex) carioca

Lane & Whitman, 1951

##### Notes

Knight and Stone 1977

#### Culex (Microculex) chryselatus

Dyar & Knab, 1919

##### Notes

Barreto-Reyes 1955

#### Culex (Microculex) daumastocampa

Dyar & Knab, 1908

##### Notes

Knight and Stone 1977

#### Culex (Microculex) elongatus

Rozeboom & Komp, 1950

##### Notes

Barreto-Reyes 1955

#### Culex (Microculex) gaudeator

Dyar and Knab, 1907

##### Notes

Gaffigan et al. 2014

#### Culex (Microculex) imitator

Theobald, 1903

##### Notes

Barreto-Reyes 1955

#### Culex (Microculex) inimitabilis

Dyar & Knab, 1906

##### Notes

Gaffigan et al. 2014

#### Culex (Microculex) jenningsi

Dyar & Knab, 1907

##### Notes

Knight and Stone 1977

#### Culex (Microculex) kukenan

Anduze, 1942

##### Notes

Knight and Stone 1977

#### Culex (Microculex) pleuristriatus

Theobald, 1903

##### Notes

Carrejo and González 1992

#### Culex (Microculex) stonei

Lane & Whitman, 1943

##### Notes

Gaffigan et al. 2014

#### Culex (Phenacomyia) airozai

Lane, 1945

##### Notes

Gaffigan et al. 2014

#### Culex (Phenacomyia) corniger

Theobald, 1903

##### Notes

Barreto-Reyes 1955

#### Culex (Phenacomyia) lactator

Dyar & Knab, 1906

##### Notes

Gaffigan et al. 2014

#### Culex (Tinolestes) latisquama

(Coquillett, 1906)

##### Notes

Carrejo and González 1992

#### Deinocerites atlanticus

Adames, 1971

##### Notes

Knight and Stone 1977

#### Deinocerites barretoi

Adames, 1971

##### Notes

Knight and Stone 1977

#### Deinocerites cancer

Theobald, 1901

##### Notes

Carrejo and González 1992

#### Deinocerites colombianus

Adames, 1971

##### Notes

Knight and Stone 1977

#### Deinocerites curiche

Adames, 1971

##### Notes

Knight and Stone 1977

#### Deinocerites dyari

Belkin & Hogue, 1959

##### Notes

Knight and Stone 1977

#### Deinocerites melanophylum

Dyar & Knab, 1907

##### Notes

Knight and Stone 1977

#### Deinocerites pseudes

Dyar & Knab, 1909

##### Notes

Knight and Stone 1977

#### Galindomyia leei

Stone & Barreto, 1969

##### Notes

Knight and Stone 1977

#### Georgecraigius (Horsfallius) fluviatilis

(Lutz, 1904)

##### Notes

Carrejo and González 1992

#### Haemagogus (Conopostegus) clarki

(Galindo, Carpenter & Trapido, 1953)

##### Notes

Knight and Stone 1977

#### Haemagogus (Conopostegus) leucocelaenus

(Dyar & Shannon, 1924)

##### Notes

Barreto-Reyes 1955

#### Haemagogus (Haemagogus) anastasionis

Dyar, 1921

##### Notes

Barreto-Reyes 1955

#### Haemagogus (Haemagogus) andinus

Osorno-Mesa, 1944

##### Notes

Barreto-Reyes 1955

#### Haemagogus (Haemagogus) boshelli

Osorno-Mesa, 1944

##### Notes

Barreto-Reyes 1955

#### Haemagogus (Haemagogus) capricornii

Lutz, 1904

##### Notes

Kumm et al. 1946. Species previously overlooked by other mosquito catalogs for Colombia.

#### Haemagogus (Haemagogus) celeste

Dyar & Nuñez Tovar, 1927

##### Notes

Knight and Stone 1977

#### Haemagogus (Haemagogus) chalcospilans

Dyar, 1921

##### Notes

Barreto-Reyes 1955

#### Haemagogus (Haemagogus) equinus

Theobald, 1903

##### Notes

Barreto-Reyes 1955

#### Haemagogus (Haemagogus) janthinomys

Dyar, 1921

##### Notes

Knight and Stone 1977

#### Haemagogus (Haemagogus) lucifer

(Howard, Dyar & Knab, 1913)

##### Notes

Barreto-Reyes 1955

#### Haemagogus (Haemagogus) spegazzinii

Brèthes, 1912

##### Notes

Barreto-Reyes 1955

#### Haemagogus (Haemagogus) splendens

Williston, 1896

##### Notes

Barreto-Reyes 1955

#### Howardina arborealis

(Bonne-Wepster & Bonne, 1920)

##### Notes

Barreto-Reyes 1955

#### Howardina eleanorae

(Berlin, 1969)

##### Notes

Knight and Stone 1977

#### Howardina fulvithorax

(Lutz, 1904)

##### Notes

Gaffigan et al. 2014

#### Howardina leei

(Berlin, 1969)

##### Notes

Knight and Stone 1977

#### Howardina marinkellei

(Berlin, 1969)

##### Notes

Knight and Stone 1977

#### Howardina osornoi

(Berlin, 1969)

##### Notes

Knight and Stone 1977

#### Howardina pseudodominicii

(Komp, 1936)

##### Notes

Knight and Stone 1977

#### Howardina quadrivittata

(Coquillett, 1902)

##### Notes

Barreto-Reyes 1955

#### Howardina septemstriata

(Dyar & Knab, 1907)

##### Notes

Barreto-Reyes 1955

#### Howardina sexlineata

(Theobald, 1901)

#### Howardina whitmorei

(Dunn, 1918)

##### Notes

Barreto-Reyes 1955

#### Isostomyia espini

(Martini, 1914)

##### Notes

Molina et al. 2000. Species previously overlooked by other mosquito catalogs for Colombia.

#### Johnbelkinia leucopus

(Dyar & Knab, 1906)

##### Notes

Molina et al. 2000. Species previously overlooked by other mosquito catalogs for Colombia.

#### Johnbelkinia longipes

(Fabricius, 1805)

##### Notes

Barreto-Reyes 1955

#### Johnbelkinia ulopus

(Dyar & Knab, 1906)

##### Notes

Knight and Stone 1977

#### Limatus asulleptus

(Theobald, 1903)

##### Notes

Barreto-Reyes 1955

#### Limatus durhami

Theobald, 1901

##### Notes

Barreto-Reyes 1955

#### Lutzia (Lutzia) allostigma

Howard, Dyar & Knab, 1915

##### Notes

Barreto-Reyes 1955

#### Lutzia (Lutzia) bigoti

Bellardi, 1862

##### Notes

Barreto-Reyes 1955

#### Mansonia (Mansonia) humeralis

Dyar & Knab, 1916

##### Notes

Barreto-Reyes 1955

#### Mansonia (Mansonia) indubitans

Dyar & Shannon, 1925

##### Notes

Ferro et al. 2008. Species previously overlooked by other mosquito catalogs for Colombia.

#### Mansonia (Mansonia) pseudotitillans

(Theobald, 1901)

##### Notes

Barreto-Reyes 1955

#### Mansonia (Mansonia) titillans

(Walker, 1848)

##### Notes

Barreto-Reyes 1955

#### Mansonia (Mansonia) wilsoni

(Barreto & Coutinho, 1944)

##### Notes

Knight and Stone 1977

#### Ochlerotatus argyrothorax

(Bonne-Wepster & Bonne, 1920)

##### Notes

Knight and Stone 1977. Species belonging to subgenus '*Protomacleaya*' *sensu auctorum*.

#### Ochlerotatus berlini

(Schick, 1970)

##### Notes

Knight and Stone 1977. Species belonging to subgenus *'*'*Protomacleaya*'*sensu auctorum*.

#### Ochlerotatus braziliensis

(Gordon & Evans, 1922)

##### Notes

Knight and Stone 1977. Species belonging to subgenus '*Protomacleaya*' *sensu auctorum*.

#### Ochlerotatus buenaventura

(Schick, 1970)

##### Notes

Knight and Stone 1977. Species belonging to subgenus *'*'*Protomacleaya*' *sensu auctorum*.

#### Ochlerotatus euiris

(Dyar, 1922)

##### Notes

Barreto-Reyes 1955. The subgeneric affiliation of this species is uncertain.

#### Ochlerotatus insolitus

(Coquillett, 1906)

##### Notes

Knight and Stone 1977. Species belonging to subgenus *'*'*Protomacleaya*' *sensu auctorum*.

#### Ochlerotatus milleri

(Dyar, 1922)

##### Notes

Barreto-Reyes 1955. The subgeneric affiliation of this species is uncertain.

#### Ochlerotatus scutellalbum

(Boshell-Manrique, 1939)

##### Notes

Barreto-Reyes 1955. The subgeneric affiliation of this species is uncertain.

#### Ochlerotatus terrens

(Walker, 1856)

##### Notes

Barreto-Reyes 1955. Species belonging to subgenus '*Protomacleaya*' *sensu auctorum*.

#### Ochlerotatus upatensis

(Anduze & Hecht, 1943)

##### Notes

Carrejo and González 1992. The subgeneric affiliation of this species is uncertain.

#### Ochlerotatus zavortinki

(Schick, 1970)

##### Notes

Knight and Stone 1977. Species belonging to subgenus *'*'*Protomacleaya*' *sensu auctorum*.

#### Ochlerotatus (Chrysoconops) fulvus

(Wiedemann, 1828)

##### Notes

Barreto-Reyes 1955

#### Ochlerotatus (Culicelsa) taeniorhynchus

(Wiedemann, 1821)

##### Notes

Barreto-Reyes 1955

#### Ochlerotatus (Ochlerotatus) angustivittatus

(Dyar & Knab, 1907)

##### Notes

Barreto-Reyes 1955

#### Ochlerotatus (Ochlerotatus) bogotanus

(Arnell, 1976)

##### Notes

Arnell 1976

#### Ochlerotatus (Ochlerotatus) comitatus

(Arnell, 1976)

##### Notes

Knight and Stone 1977

#### Ochlerotatus (Ochlerotatus) condolescens

(Dyar & Knab, 1907)

##### Notes

Knight and Stone 1977

#### Ochlerotatus (Ochlerotatus) crinifer

(Theobald, 1903)

##### Notes

Barreto-Reyes 1955

#### Ochlerotatus (Ochlerotatus) deficiens

(Arnell, 1976)

##### Notes

Knight and Stone 1977

#### Ochlerotatus (Ochlerotatus) euplocamus

(Dyar & Knab, 1906)

##### Notes

Knight and Stone 1977

#### Ochlerotatus (Ochlerotatus) pectinatus

(Arnell, 1976)

##### Notes

Knight and Stone 1977

#### Ochlerotatus (Ochlerotatus) scapularis

(Rondani, 1848)

##### Notes

Barreto-Reyes 1955

#### Ochlerotatus (Protoculex) aenigmaticus

(Cerqueira & Costa, 1946)

##### Notes

Gaffigan et al. 2014

#### Ochlerotatus (Protoculex) hastatus

(Dyar, 1922)

##### Notes

Barreto-Reyes 1955

#### Ochlerotatus (Protoculex) nubilus

(Theobald, 1903)

##### Notes

Carrejo and González 1992

#### Ochlerotatus (Protoculex) serratus

(Theobald, 1901)

##### Notes

Barreto-Reyes 1955

#### Onirion personatum

(Lutz, 1904)

##### Notes

Knight and Stone 1977

#### Orthopodomyia albicosta

(Lutz, 1904)

##### Notes

Barreto-Reyes 1955

#### Orthopodomyia fascipes

(Coquillett, 1906)

##### Notes

Barreto-Reyes 1955

#### Orthopodomyia phyllozoa

(Dyar & Knab, 1907)

##### Notes

Barreto-Reyes 1955

#### Psorophora (Grabhamia) cingulata

(Fabricius, 1805)

##### Notes

Barreto-Reyes 1955

#### Psorophora (Grabhamia) confinnis

(Lynch Arribálzaga, 1891)

##### Notes

Barreto-Reyes 1955

#### Psorophora (Janthinosoma) albigenu

(Peryassú, 1908)

##### Notes

Knight and Stone 1977

#### Psorophora (Janthinosoma) albipes

(Theobald, 1907)

##### Notes

Barajas et al. 2013

#### Psorophora (Janthinosoma) cyanescens

(Coquillett, 1902)

##### Notes

Barreto-Reyes 1955

#### Psorophora (Janthinosoma) ferox

(von Humboldt, 1819)

##### Notes

Barreto-Reyes 1955

#### Psorophora (Janthinosoma) lutzi

(Theobald, 1901)

##### Notes

Barreto-Reyes 1955

#### Psorophora (Janthinosoma) pilosa

Duret, 1971

##### Notes

Knight and Stone 1977

#### Psorophora (Janthinosoma) pseudoalbipes

Duret, 1971

##### Notes

Knight and Stone 1977

#### Psorophora (Janthinosoma) varipes

(Coquillett, 1904)

##### Notes

Barreto-Reyes 1955

#### Psorophora (Psorophora) ciliata

(Fabricius, 1794)

##### Notes

Barreto-Reyes 1955

#### Psorophora (Psorophora) cilipes

(Fabricius, 1805)

##### Notes

Barreto-Reyes 1955

#### Psorophora (Psorophora) lineata

(von Humboldt, 1819)

##### Notes

Barreto-Reyes 1955

#### Psorophora (Psorophora) saeva

Dyar & Knab, 1906

##### Notes

Liria and Navarro 2003. Species previously overlooked by other mosquito catalogs for Colombia.

#### Runchomyia (Ctenogoeldia) magna

(Theobald, 1905)

##### Notes

Knight and Stone 1977

#### Sabethes (Peytonulus) identicus

Dyar & Knab, 1907

##### Notes

Gaffigan et al. 2014

#### Sabethes (Peytonulus) ignotus

Harbach, 1995

##### Notes

Harbach 1995

#### Sabethes (Peytonulus) undosus

(Coquillett, 1906)

##### Notes

Carrejo and González 1992

#### Sabethes (Peytonulus) xenismus

Harbach, 1995

##### Notes

Harbach 1995

#### Sabethes (Sabethes) albiprivus

Theobald, 1903

##### Notes

Barreto-Reyes 1955

#### Sabethes (Sabethes) belisarioi

Neiva, 1908

##### Notes

Barreto-Reyes 1955

#### Sabethes (Sabethes) cyaneus

(Fabricius, 1805)

##### Notes

Barreto-Reyes 1955

#### Sabethes (Sabethes) quasicyaneus

Peryassú, 1922

##### Notes

Knight and Stone 1977

#### Sabethes (Sabethes) tarsopus

Dyar & Knab, 1908

##### Notes

Barreto-Reyes 1955

#### Sabethes (Sabethinus) intermedius

(Lutz, 1904)

##### Notes

Barreto-Reyes 1955

#### Sabethes (Sabethoides) chloropterus

(von Humboldt, 1819)

##### Notes

Barreto-Reyes 1955

#### Sabethes (Sabethoides) glaucodaemon

(Dyar and Shannon, 1925)

##### Notes

Molina et al. 2000. Species previously overlooked by other mosquito catalogs for Colombia.

#### Sallumia hortator

(Dyar & Knab, 1907)

##### Notes

Knight and Stone 1977

#### Shannoniana fluviatilis

(Theobald, 1903)

##### Notes

Barreto-Reyes 1955

#### Stegomyia albopicta

(Skuse, 1895)

##### Notes

Vélez et al. 1998. The subgeneric affiliation of this species is uncertain.

#### Stegomyia (Stegomyia) aegypti

(Linnaeus, 1762)

##### Notes

Knight and Stone 1977

#### Toxorhynchites (Lynchiella) bambusicola

(Lutz & Neiva, 1913)

##### Notes

Barreto-Reyes 1955

#### Toxorhynchites (Lynchiella) guadeloupensis

(Dyar & Knab, 1906)

##### Notes

Knight and Stone 1977

#### Toxorhynchites (Lynchiella) haemorrhoidalis

(Fabricius, 1787)

##### Notes

Knight and Stone 1977

#### Toxorhynchites (Lynchiella) hypoptes

(Knab, 1907)

##### Notes

Knight and Stone 1977

#### Toxorhynchites (Lynchiella) moctezuma

(Dyar & Knab, 1906)

##### Notes

Gaffigan et al. 2014

#### Toxorhynchites (Lynchiella) theobaldi

(Dyar & Knab, 1906)

##### Notes

Barreto-Reyes 1955

#### Trichoprosopon andinum

Levi-Castillo, 1953

##### Notes

Carrejo and González 1992

#### Trichoprosopon compressum

Lutz, 1905

##### Notes

Barreto-Reyes 1955

#### Trichoprosopon digitatum

(Rondani, 1848)

##### Notes

Barreto-Reyes 1955

#### Trichoprosopon evansae

Antunes, 1942

##### Notes

Barreto-Reyes 1955

#### Trichoprosopon lanei

(Antunes, 1937)

##### Notes

Barreto-Reyes 1955

#### Trichoprosopon pallidiventer

(Lutz, 1905)

##### Notes

Barreto-Reyes 1955

#### Uranotaenia (Uranotaenia) apicalis

Theobald, 1903

##### Notes

Carrejo and González 1992

#### Uranotaenia (Uranotaenia) calosomata

Dyar & Knab, 1907

##### Notes

Barreto-Reyes 1955

#### Uranotaenia (Uranotaenia) coatzacoalcos

Dyar & Knab, 1906

##### Notes

Barreto-Reyes 1955

#### Uranotaenia (Uranotaenia) geometrica

Theobald, 1901

##### Notes

Barreto-Reyes 1955

#### Uranotaenia (Uranotaenia) hystera

Dyar & Knab, 1913

##### Notes

Barreto-Reyes 1955

#### Uranotaenia (Uranotaenia) leucoptera

(Theobald, 1907)

##### Notes

Carrejo and González 1992

#### Uranotaenia (Uranotaenia) lowii

Theobald, 1901

##### Notes

Barreto-Reyes 1955

#### Uranotaenia (Uranotaenia) pulcherrima

Lynch Arribálzaga, 1891

##### Notes

Barreto-Reyes 1955

#### Uranotaenia (Uranotaenia) riverai

Duret, 1970

##### Notes

Knight and Stone 1977

#### Wyeomyia chalcocephala

Dyar & Knab, 1906

##### Notes

Knight and Stone 1977. The subgeneric affiliation of this species is uncertain.

#### Wyeomyia clasoleuca

Dyar & Knab, 1908

##### Notes

Barreto-Reyes 1955. The subgeneric affiliation of this species is uncertain.

#### Wyeomyia melanocephala

Dyar & Knab, 1906

##### Notes

Barreto-Reyes 1955. The subgeneric affiliation of this species is uncertain.

#### Wyeomyia moerbista

(Dyar & Knab, 1919)

##### Notes

Barreto-Reyes 1955. The subgeneric affiliation of this species is uncertain.

#### Wyeomyia serratoria

(Dyar & Nunez Tovar, 1927)

##### Notes

Barreto-Reyes 1955. The subgeneric affiliation of this species is uncertain.

#### Wyeomyia (Antunesmyia) colombiana

Lane, 1945

##### Notes

Barreto-Reyes 1955

#### Wyeomyia (Antunesmyia) flavifacies

Edwards, 1922

##### Notes

Barreto-Reyes 1955

#### Wyeomyia (Cruzmyia) kummi

Lane & Cerqueira, 1942

##### Notes

Knight and Stone 1977

#### Wyeomyia (Cruzmyia) mattinglyi

Lane, 1953

##### Notes

Knight and Stone 1977

#### Wyeomyia (Decamyia) ulocoma

(Theobald, 1903)

##### Notes

Carrejo and González 1992

#### Wyeomyia (Decamyia) pseudopecten

Dyar & Knab, 1906

##### Notes

Barreto-Reyes 1955

#### Wyeomyia (Dendromyia) complosa

(Dyar, 1928)

##### Notes

Barreto-Reyes 1955

#### Wyeomyia (Dendromyia) jocosa

(Dyar & Knab, 1908)

##### Notes

Knight and Stone 1977

#### Wyeomyia (Dendromyia) luteoventralis

Theobald, 1901

##### Notes

Species newly recorded for Colombia (see Material & methods for detailed locality information).

#### Wyeomyia (Dendromyia) ypsipola

Dyar, 1922

##### Notes

Barreto-Reyes 1955

#### Wyeomyia (Dodecamyia) aphobema

Dyar, 1918

##### Notes

Barreto-Reyes 1955

#### Wyeomyia (Exallomyia) tarsata

Lane & Cerqueira, 1942

##### Notes

Knight and Stone 1977

#### Wyeomyia (Hystatomyia) chocoensis

Porter & Wolff, 2004

##### Notes

Porter and Wolff 2004

#### Wyeomyia (Hystatomyia) intonca

Dyar & Knab, 1910

##### Notes

Porter and Wolff 2004. Species previously overlooked by other mosquito catalogs for Colombia.

#### Wyeomyia (Miamyia) codiocampa

Dyar & Knab, 1906

##### Notes

Carrejo and González 1992

#### Wyeomyia (Miamyia) hosautos

Dyar & Knab, 1907

##### Notes

Barreto-Reyes 1955

#### Wyeomyia (Miamyia) oblita

(Lutz, 1904)

##### Notes

Barajas et al. 2013

#### Wyeomyia (Triamyia) aporonoma

Dyar & Knab, 1906

##### Notes

Barreto-Reyes 1955

#### Wyeomyia (Wyeomyia) arthrostigma

(Lutz, 1905)

##### Notes

Carrejo and González 1992

#### Wyeomyia (Wyeomyia) celaenocephala

Dyar & Knab, 1906

##### Notes

Barreto-Reyes 1955

#### Wyeomyia (Wyeomyia) melanopus

Dyar, 1919

##### Notes

Carrejo and González 1992

#### Wyeomyia (Wyeomyia) pertinans

(Williston, 1896)

##### Notes

Carrejo and González 1992

#### Wyeomyia (Wyeomyia) scotinomus

(Dyar & Knab, 1907)

##### Notes

Barreto-Reyes 1955

#### Wyeomyia (Wyeomyia) simmsi

(Dyar & Knab, 1908)

##### Notes

Carrejo and González 1992

## Discussion

This paper comprises the most complete and up-to-date list of mosquito species known to occur in Colombia, with a total of 324 species and 28 genera listed according to the latest nomenclature and classification. Our systematic review and literature survey found, by 16 February 2015, 13 records of culicid speciesoverlooked by previous mosquito catalogs for Colombia.​ These are: *Anopheles costai*, *An. fluminensis* and *An. shannoni* (cited by González and Carrejo 2007), *An. malefactor* (cited by Wilkerson 1990), *An. vargasi* (cited by Osorno-Mesa 1947), *Culex mesodenticulatus* (cited by Barreto et al. 1996), *Haemagogus capricornii* (cited by Kumm et al. 1946), *Isostomyia espini*, *Johnbelkinia leucopus* and *Sabethes glaucodaemon* (cited by Molina et al. 2000), *Mansonia indubitans* (cited by Ferro et al. 2008), *Psorophora saeva* (cited by Liria and Navarro 2003 and *Wyeomyia intonca* (cited by Porter and Wolff 2004).

In addition, we record Wyeomyia (Dendromyia) luteoventralis for the first time from Colombia. This species was originally described by Theobald (1901) from three females caught on human bait in Paraná state (Brazil). *Wyeomyia luteoventralis* is the type species of the subgenus *Dendromyia* Theobald, 1903 (Motta and Lourenço-de-Oliveira 1995). This species had been previously collected in Brazil, French Guiana, British Guiana, and Venezuela (Gaffigan et al. 2014); however, it has rarely been reported in biological, ecological and taxonomic studies in South America (Motta and Lourenço-de-Oliveira 1995).

Although this report represents the most comprehensive list of mosquito species for Colombia, the 324 species included in this updated listing are unlikely to be a complete inventory of the Colombian mosquito fauna. Biodiversity data suggest that around 1,000 mosquito species will eventually be described from southern U.S.A. to Colombia, Ecuador, and Peru (Belkin et al. 1965, Foley et al. 2008). Colombia, with its diversity of landscapes, is highly species-rich and is a potential hotspot of mosquito endemism (Foley et al. 2008). Furthermore, ongoing and rapid development in Colombia is expected in the next decade with a loss of habitat associated with the growth of urban centers, which will undoubtedly have an impact on species diversity (Montoya-Lerma et al. 2011). Moreover, the finding of new species to the Colombian fauna such as *Wyeomyia luteoventralis* indicates that future sampling may reveal further species records which could help improve the taxonomy and provide a more accurate estimate of Colombia's mosquito diversity. This work provides important insights into the mosquito diversity of Colombia. We expect it to serve as a useful platform for future work on the mosquitoes of Colombia as well as provide a context for non-vector species.
